# Reach and impact of a nationwide media campaign in Ethiopia for promoting safe breastfeeding practices in the context of the COVID-19 pandemic

**DOI:** 10.1186/s44263-024-00065-2

**Published:** 2024-06-10

**Authors:** Abel Negussie, Bereket Tefera, Elyas Melaku Mazengia, Ariam Hailemariam, Ephrem Lejore, Tariku Dejene, Abiy Tefera, Ramadhani Noor, Stanley Chitekwe, Hiwot Getachew, Rachana Sharma, Eshetu Girma

**Affiliations:** 1 Department of Social and Population Health, Yirgalem Hospital Medical College, Yirgalem, Ethiopia; 2 School of Public Health, College of Medicine and Health Sciences, Dire Dawa University, Dire Dawa, Ethiopia; 3 School of Public Health, College of Health Sciences, Debre Markos University, Debre Markos, Ethiopia; 4 School of Medicine, College of Health Sciences, Addis Ababa University, Addis Ababa, Ethiopia; 5 School of Public Health, College of Medicine and Health Sciences, Hawassa University, Hawassa, Ethiopia; 6 Center for Population Studies, College of Development Studies, Addis Ababa University, Addis Ababa, Ethiopia; 7 Nutrition Section, United Nations Children’s Fund, Addis Ababa, Ethiopia; 8 Department of Global Health and Population, Harvard T. H. Chan School of Public Health, Boston, MA USA; 9 Social and Behavior Change (SBC) Section, United Nations Children’s Fund, Addis Ababa, Ethiopia; 10 School of Public Health, College of Health Sciences, Addis Ababa University, Addis Ababa, Ethiopia; 11 Ethiopian Health Education and Promotion Professionals Association (EHEPA), Addis Ababa, Ethiopia

**Keywords:** Safe breastfeeding, COVID-19 pandemic, Media campaign, Reach, Impact, Mobile survey, Ethiopia

## Abstract

**Background:**

In response to the COVID-19 challenge and the consequent concerns and misconceptions about potential mother-to-child virus transmission, the United Nations Children’s Fund (UNICEF), in collaboration with the Ethiopian Ministry of Health, launched a 3-month nationwide media campaign to promote appropriate and safe breastfeeding practices using national and regional television and radio channels, as well as social media. This study assesses the reach and impact of a media campaign in Ethiopia on improving mothers’, partners’/caregivers’, and the public’s awareness of and practices related to appropriate and safe breastfeeding.

**Methods:**

A two-round mobile survey was conducted using random digit dialing (RDD) and an interactive voice response (IVR) system. In order to assess the impact of the media campaign, the study compared outcomes related to awareness, perceptions, and safe breastfeeding practices using post-intervention comparison data across levels of exposure (exposed vs. unexposed to the campaign). A propensity score matching (PSM) analysis was performed using a two sample test of proportions to estimate the impact of the media campaign.

**Results:**

Among the 3170 mobile subscribers who completed the survey questions, half (50%) reported that they had seen or heard media advertisements about appropriate breastfeeding, the importance of continuing breastfeeding during the COVID-19 pandemic, and how to safely breastfeed a baby when a mother is suspected or confirmed with COVID-19. The PSM analysis showed that exposure to the media campaign was significantly associated with awareness and perceptions of the importance of continuing appropriate breastfeeding during the pandemic among the general public (proportion difference, 0.16; 95% CI, 0.12–0.19; *p* < 0.0001) and mothers with children under 2 years old or their partners (proportion difference, 0.06; 95% CI, 0.01–0.12, *p* = 0.01).

**Conclusions:**

The nationwide media campaign promoting safe breastfeeding practices in the context of COVID-19 reached half of the target breastfeeding mothers and the general public and had a significant impact on awareness and perceptions about the importance of continuing appropriate and safe breastfeeding practices. Future media campaigns should ensure that the intensity and frequency of media spots are appropriate to achieve adequate exposure, message recall, and influence infant and young child feeding behaviors.

**Supplementary Information:**

The online version contains supplementary material available at 10.1186/s44263-024-00065-2.

## Background

Although there is a global recognition that the first 1000 days of life are critical for ensuring optimal growth and development [1] and evidence-based practices for infant and young child feeding (IYCF) have been identified [2], mothers/caregivers in low- and middle-income countries (LMICs) may employ inappropriate IYCF practices, exposing children to high risk of malnutrition, growth faltering, and stunting [3, 4]. Global estimates indicate that 45 million children under the age of five were wasted and 149 million were stunted in 2020, from which Asia and Africa have the greatest burden of global cases of stunting and wasting [5]. In Ethiopia, as well, poor feeding practices during infancy and early childhood are the major causes of childhood undernutrition [6]; and the prevalence of early initiation of breastfeeding and exclusive breastfeeding was estimated to be 75.5% and 59.9%, respectively [7].

The COVID-19 pandemic has affected many LMICs, including sub-Saharan African countries, exacerbating social and economic risks that may pose a major danger to infant and child nutrition status and feeding practices [8–10]. Appropriate IYCF practices are important not only during normal times but also during a pandemic to protect the health of the babies. The guiding recommendations made by the World Health Organization (WHO) [11] and United Nations Children’s Fund (UNICEF) [12] is that mothers should continue appropriate breastfeeding and complementary child feeding practices as before the COVID-19 pandemic, which include exclusive breastfeeding until 6 months of age even in cases where the mother has suspected or confirmed COVID-19 infection while taking necessary precautions, and the provision of safe and nutrient-rich foods in sufficient amounts, in addition to breast milk, from 6 to 23 months of age. These recommendations are also supported by existing evidence and balance of risk estimates that show how newborn babies and infants, especially in LMICs, could suffer severe consequences if proper IYCF practices are not sustained during the COVID-19 pandemic situation [10, 13, 14]. However, different studies revealed that mothers during the COVID-19 pandemic had concerns and misconceptions about IYCF practices [9, 15, 16]. For instance, a study conducted in Thailand during the COVID-19 lockdown showed that mothers decreased breastfeeding their infants [17]. Therefore, the global response to the COVID-19 pandemic needs to integrate and respond to issues with under-five child nutrition and promote appropriate IYCF knowledge and practices through community-wide interventions [9].

In the context of COVID-19, providing accurate information on optimal and safe breastfeeding practices to mothers and the general public using locally appropriate media channels is especially essential to clarify potential knowledge, perceptions, and confidence-related barriers as well as address specific misconceptions and misinformation concerning COVID-19 and breastfeeding practices. Research shows that media campaigns can be effective to promote health behaviors, including IYCF behaviors [18–24]. Also, studies revealed that media exposure has influenced COVID-19-related preventive practices (e.g., generating demand for COVID-19 vaccination), raising public awareness, and overcoming misinformation and infodemics (the spread of excessive health information, accurate or not, during the COVID-19 pandemic) [25–28]. Consequently, mass media campaigns that are matched to the media preferences of particular population groups and contexts can be effective strategies for communicating behavior change messages. They can positively impact IYCF behaviors due to their potential advantage of reaching a large proportion of mothers and others who influence their IYCF practices at a relatively low cost, control over message content and delivery, and ease of translation into multiple languages [18, 21, 24, 29–32].

In 2020, UNICEF and the Ministry of Health of Ethiopia launched a nationwide media campaign using the most popular national and regional television and radio channels in the country, as well as social media (Facebook and YouTube), to promote mothers’ exclusive breastfeeding and safe breastfeeding-related information and practices during the COVID-19 pandemic. This study evaluates the campaigns’ reach and potential impact on public awareness of appropriate and safe breastfeeding behaviors in order to provide insight into how, why, and what specific aspects of the campaign have worked or not. Additionally, the study’s findings can be extended to other situations beyond the COVID-19 pandemic context, where media campaigns can be introduced to communicate messages to the general public in order to quickly dispel myths and misbeliefs that are circulating during uncertain emergency environments and when there is lack of credible and easily accessible health information. Such evaluation studies can also help to learn lessons for effective use of future media campaigns that promote optimal IYCF practices [21, 33–35].

Furthermore, in Ethiopia and other resource-limited settings, suboptimal and inappropriate feeding and breastfeeding practices are substantial among mothers exposed to infectious diseases such as HIV [36–41], and there is sparse evidence on the potential contribution of media campaigns to address mothers’ concerns about vertical infection transmission and promote appropriate breastfeeding practices. Previous research indicated that efforts to promote breastfeeding among mothers exposed to infectious diseases, including HIV, used intervention strategies such as providing education and counseling on recommended infant feeding options during antenatal and postnatal care visits, psychosocial and family/peer support, and personalized home-based counseling by community health volunteers [42–45]. On top of current efforts, media campaign strategies can support large-scale community education to promote appropriate breastfeeding practices, particularly among women with infectious diseases. In this regard, the findings of the campaign’s evaluation can serve to inform what better media intervention strategies can be used to promote safe breastfeeding practices for mothers who are exposed to infectious diseases. The aim of the present study was to evaluate the level of exposure or reach and the impact of the media campaign on improving mothers’, caregivers’, and the public’s awareness of and practices about safe breastfeeding in the context of COVID-19. The evaluation data was supposed to explore the following:

1. Reach: How wide is the reach of the media campaign, and what proportion of the target audience recalls the key campaign messages? Does the reach of the media campaign vary across regions and socio-demographic characteristics?
2. Impact of the media campaign on safe breastfeeding-related awareness and perceptions of mothers, their partners, and the general public: Can we link exposure to the campaign to appropriate breastfeeding behaviors? Did the media campaigns contribute for behavior change?

### Methods

#### Study context and description of the media campaign

In response to the COVID-19 pandemic in Ethiopia, UNICEF/Ethiopia in collaboration with the Federal Ministry of Health conducted nationwide media campaign for 3 months (from August 1 to October 31, 2020) to promote appropriate and safe breastfeeding practices. The purpose of the media campaign was to improve the awareness and behavior of mothers of children aged 0–23 months and the general public about appropriate breastfeeding, the importance of continuing breastfeeding, and the use of safe breastfeeding practices when the mother is suspected or confirmed with COVID-19. More specifically, the campaign included key messages on the importance of continuing appropriate breastfeeding for at least two years, how a mother suspected or confirmed to have COVID-19 can breastfeed her child while applying precautions, and how a seriously ill mother can express breast milk and use safe breastfeeding practices.

Sixty-second spots—short recorded adverts that appear in a radio or television commercial schedule—were broadcast on ten and eight popular national and regional TV and radio channels, respectively, in six different local languages along with sign language. A total of 48 spots (1 spot per day × 4 times per week × 12 weeks) were aired on almost all TV and radio channels, and prime times were selected to transmit the messages. Additionally, social media was used to disseminate the same message. The video spot was posted on the official UNICEF Ethiopia Facebook page, with a reach of 3249 people and 670,545 impressions. The number of times people viewed the video for at least 3 and 15 seconds was 203,055 and 54,374, respectively. The video post received 2600 likes, 567 shares, and 44 comments during the campaign. Engagement was comparable between males and females (51% women, 49% men), but it was higher in the 25**–**34 and 35**–**44 age categories. At the end of the campaign, two rounds of mobile surveys via interactive voice response (IVR) were conducted to assess its reach and impact.

#### Evaluation design

A post-comparison group evaluation design was employed. The evaluation study compared outcomes using post-intervention comparison data across levels of exposure (exposed vs. unexposed to the campaign) to assess the impact of the media campaign on awareness, perceptions, and safe breastfeeding-related behaviors.

It was not possible to establish a control group that was not exposed to the intervention because the media campaign implementation had a national scope. To compensate for the lack of a control group, a post-comparison group (i.e., participants who were not exposed to the campaign messages) was created in order to compare proper breastfeeding awareness and practices between study participants who had been exposed to the media messages and those who had not, as well as to identify associations between exposure and appropriate breastfeeding practices among mothers who had been with suspected or confirmed COVID-19 illness.

#### Selection of study participants

Random digit dialing (RDD) panels that target all mobile network subscribers and IVR systems were applied to randomly select and call a mobile number and automatically interact with the participant in the mobile survey. With the added benefit of including unlisted numbers that would be missed if the numbers were selected from a phone book, RDD is a method for selecting participants in phone surveys. The targeted number of complete responses was 3000. This sample size was large enough to give statistically valid estimates.

Mothers of children aged 0 to 23 months were the target audience for the media messages. However, mothers’ partners/fathers, as well as the general public, were also eligible to participate in the mobile survey in order to assess the reach and impact of the media messages, since they may be potential sources of information and influence mothers’ breastfeeding practices.

#### Data collection and measurements

Viamo, a global social enterprise experienced in a mobile survey platform [46], performed a two-round, nationwide mobile survey in six languages: Amharic, Afan Oromo, Tigrigna, Somali, Afar, and Nuer. The first-round survey data was collected over a three-week period from November 9 to November 29, 2020, and the second-round survey data was collected from January 21 to March 13, 2021. The mobile survey instrument was designed in a series of steps. Viamo/Ethiopia first developed mobile-friendly content to understand the knowledge, perception, and behavior of the target users. The developed content was then optimized through consultation with the UNICEF/Ethiopia Nutrition program team experts based on the key messages of the media campaign. Following that, the content was designed on the Viamo platform with the necessary quality checks including both internal and external tests. Content of the survey was launched, and calls were made to each subscriber to a mobile network using an RDD pattern. If a respondent missed the call and wanted to participate in the survey or not, they could call back. One to three call attempts were made per number. The system included a language selector, an introduction explaining the source and purpose of the call as well as a brief sensitization on how to answer questions on their phones.

Participants were asked about the importance of maintaining breastfeeding during the COVID-19 pandemic, what to do if a breastfeeding mother has COVID-19 and what precautions she should take, and what a mother should do when she is seriously ill and unable to breastfeed. The knowledge on breastfeeding was deemed correct when a participant mentioned the importance of continuing breastfeeding (i.e., mothers should continue appropriate breastfeeding as before the COVID-19 pandemic, which include exclusive breastfeeding until six months of age) and was aware of proper and safe breastfeeding practices in cases where the mother has been suspected or confirmed to have COVID-19 and/or is severely sick to continue breastfeeding in accordance with the advised practices (i.e., continue breastfeeding while taking necessary precautions, and the provision of safe and nutrient-rich foods in sufficient amounts, in addition to breast milk, for children aged 6 to 23 months) as promoted in the media advertisements.

Awareness and practices of appropriate and safe breastfeeding were the target outcome variables. Exposure to the media campaign was measured using a combination of self-reported exposure to either of the media channel messages and recall of at least one of the campaign’s key message contents in the media spots (the importance of continuing breastfeeding during the COVID-19 pandemic, what a breastfeeding mother with COVID-19 symptoms should do and what precautions she should take, and what a mother should do when she is seriously ill and unable to breastfeed). Covariates included sociodemographic characteristics including age, gender, and place of residence (region, urban or rural residence).

#### Data analysis

Using tables and selected figures, basic descriptive statistics were presented, including a summary of the media campaign’s reach. Additionally, the survey data was used to compare media exposure status by sociodemographic characteristics. Key impact indicator percentages were calculated, and exposure to media spots (i.e., self-report and recall of at least one of the campaign’s specific message vs. unexposed or did not recall) was also compared to awareness and perceptions about appropriate and safe breastfeeding practices when a mother is suspected or confirmed with COVID-19.

In order to assess the impact of a media campaign on awareness and perceptions of appropriate breastfeeding when a mother is suspected or confirmed with COVID-19, a propensity score matching (PSM) analysis was conducted. PSM is a statistical technique commonly used in observational studies to reduce bias and mimic the randomization process of a randomized controlled trial when randomization is not possible [47]. It helps to create a balanced comparison group by matching individuals based on their propensity scores, which are estimated probabilities of receiving the treatment (in this case, exposure to the media campaign). The method used in the analysis employed the nearest-neighbor method with one-to-one matching. This approach involves identifying individuals in the control group who have similar propensity scores to those in the exposed group [48]. The propensity score is the estimated probability of being exposed to the media campaign, based on the potential confounder variables included in the propensity score model. In this study, the potential confounder variables included in the propensity score model were region, age, gender, and residence. By including these variables in the model, the researchers aimed to create a balanced comparison group that closely resembled the exposed group in terms of these characteristics. PSM results were demonstrated as proportion difference (PD) and 95% confidence intervals (CI), and analyses were performed using Stata version 17.

In PSM analysis, common support is important because it ensures that there is a sufficient number of treated and control units with similar propensity scores, allowing for meaningful comparisons. Without common support, it becomes challenging to find suitable matches for all treated units, which can compromise the validity of the analysis [48, 49]. In the present analysis, we find that the groups are comparable, and there were no off-support observations (see Additional file 1: Fig. S1). After performing the PSM, the balance of covariates between the exposed and control groups was also assessed using covariate imbalance test. This was done to ensure that the matching process had effectively balanced the distribution of the potential confounder variables. The results showed that the matching resulted in a reduction of an average of 12**–**18% bias. The balance between the propensity scores before and after match were also compared. The matching for both the total sample and mothers with under two years child resulted in a balanced propensity score after matching with a 42% and 44% bias reduction, respectively.

## Results

### Overall response to the mobile survey

A total of 163,578 calls were pushed out to subscribers, and 21,307 unique subscribers responded, and 8416 (39.5%) of the respondents were call-backs. Of the 5976 mobile subscribers who answered the consent question, 3170 (53%) agreed to participate in the study and completed the survey questions. The mean call duration of the completed survey calls was 377.5 (standard deviation of 124) seconds.

### Sociodemographic characteristics of participants

Most of the participants were from the Oromia region (41.4%), followed by Addis Ababa (22.5%) and Amhara (19.7%). In addition, more than three-quarters (78.6%) of the participants were male, and 81% of them were between the ages of 18 and 34. The majority of participants (72.2%) live in urban areas, and one-third of participants (33.3%) had children under the age of 2. Even though 21,307 subscribers who answered the survey calls indicated their preference for all six languages, only three languages (Amharic, Afan Oromo, and Somali) were actually used by the participants to complete the survey and provide their responses. A detailed summary of the participants’ sociodemographic profile is presented in Table 1.

**Table 1:** Sociodemographic profile of the study participants who completed the survey, *N* = 3170

Participant characteristics	Number of participants, *n* (%)
**Age (years)**	
18–24	1393 (43.9%)
25–34	1168 (36.8%)
35–44	452 (14.2%)
45 and above	157 (4.9%)
**Gender**	
Male	2493 (78.6%)
Female	677 (21.3%)
**Region**	
Oromia	1312 (41.3%)
Addis Ababa	653 (20.5%)
Amhara	623 (19.6%)
Southern Nations, Nationalities, and Peoples (SNNP)	327 (10.3%)
Somali	85 (2.6%)
Other region	170 (5.2%)
**Children < 2 years of age**	
Yes	1057 (33.3%)
No	2113 (66.7%)
**Language selected to answer survey questions**	
Amharic	2241 (70.6%)
Afan Oromo	834 (26.3%)
Somali	92 (2.9%)
**Residence**	
Urban	2290 (72.2%)
Peri-urban	499 (15.7%)
Rural	381 (12%)

### Reach of the media campaign

**Fig. 1:**
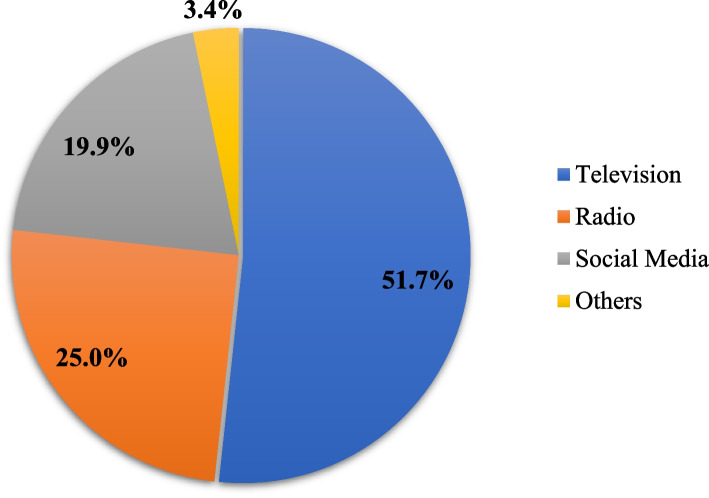
Main source of information to the media campaign promoting safe breastfeeding practices in the context of COVID-19, 2020

Participants were asked if they had seen or heard any media advertisements about the importance of continuing breastfeeding and how to safely breastfeed a baby during the pandemic, and 1576 (49.7%) of them reported they had, whereas 1594 (50.3%) reported they had not. According to 815 (51.7%) of the respondents, television was the most common source of information (Fig. 1). Furthermore, the participants were asked if they had learned anything new from the spots they had accessed, and 1344 (85.2%) responded that they had. Among them, 229 (17%) learned the importance of continuing breastfeeding, and 517 (38.5%) learned how to breastfeed a child when a mother is suspected or confirmed with COVID-19.

The media campaign reach was also analyzed by sociodemographic variables, particularly among target mothers (or their partners/fathers) with under-two-year-old children. As a result, a total of 1057 (33.3%) survey participants had children under the age of two. Of them, 190 (18%) were mothers, and 721 (68.2%) were urban residents. More than half of the respondents (51.5%) with children under two years old had heard or seen the campaign advertising, with 110 (21%) of them being mothers, and television was the dominant information source (Table 2). Moreover, the level of exposure to the media ads among Addis Ababa participants was found to be higher than the total average level of exposure (67.7%), and only nearly one-fourth of the rural participants were exposed to the campaign messages.

**Table 2:** Exposure level to the nationwide media campaign ads promoting appropriate and safe breastfeeding practices in the context of COVID-19, by sociodemographic variables, among participants with children under two years old, 2020, *n* = 1057

Variables	Total number of participants with children < 2 years old *n* (%)	Participants with children < 2 years old exposed to the media ads *n* (%)	Main source of information				
			**Television** ***n* (%)**	**Radio** ***n* (%)**	**Social media** ***n* (%)**	**Other** ***n* (%)**	**Total** ***n* (%)**
**Region**							
Oromia	507 (47.9)	256 (50.4)	118 (46)	88 (34.3)	26 (10.1)	24 (9.3)	256 (100)
Addis Ababa	180 (17)	122 (67.7)	59 (48.3)	19 (15.5)	31 (25.4)	13 (10.6)	122 (100)
Amhara	99 (9.3)	80 (44.4)	38 (47.5)	24 (30)	13 (16.2)	5 (6.2)	80 (100)
SNNP	180 (17)	45 (45.4)	25 (55.5)	7 (15.5)	6 (13.3)	7 (15.5)	45 (100)
Other region	91 (8.6)	42 (46.1)	11 (26.1)	11 (26.1)	13 (30.9)	7 (16.6)	42 (100)
Total	1,057 (100)	545 (51.5)	251 (46)	149 (27.3)	89 (16.3)	56 (10.2)	545 (100)
**Gender**							
Male	867 (82)	435 (50.1)	190 (43.6)	127 (29.1)	74 (17)	44 (10.1)	435 (100)
Female	190 (17.9)	110 (57.8)	61 (55.4)	22 (20)	15 (13.6)	12 (10.9)	110 (100)
Total	1,057 (100)	545 (51.5)	251 (46)	149 (27.3)	89 (16.3)	56 (10.2)	545 (100)
**Residence**							
Urban	721 (68.2)	413 (58.2)	209 (50.6)	94 (22.7)	71 (17.1)	39 (9.4)	413 (100)
Peri-urban	177 (14)	73 (68.3)	28 (38.3)	25 (34.2)	9 (12.3)	11 (15)	73 (100)
Rural	159 (9.2)	59 (24.3)	14 (23.7)	30 (50.8)	9 (15.2)	6 (10.1)	59 (100)
Total	1,057 (100)	545 (51.5)	251 (46)	149 (27.3)	89 (16.3)	56 (10.2)	545 (100)
**Age (years)**							
18–24	456 (43.1)	214 (46.9)	71 (33.1)	93 (43.4)	24 (11.2)	26 (12.1)	214 (100)
25–34	427 (40.3)	231 (54)	122 (52.8)	40 (17.3)	48 (20.7)	21 (9)	231 (100)
35–44	148 (14)	88 (59.4)	53 (60.2)	14 (15.9)	16 (18.1)	5 (5.6)	88 (100)
45 and above	26 (2.4)	12 (46.1)	5 (41.6)	2 (16.6)	1 (8.3)	4 (33.3)	12 (100)
Total	1,057 (100)	545 (51.5)	251 (46)	149 (27.3)	89 (16.3)	56 (10.2)	545 (100)

### Impact on awareness, public perception, and safe breastfeeding practices

To assess whether the media campaign has influenced awareness and perceptions of the importance of maintaining appropriate breastfeeding practices during the COVID-19 pandemic, participants were asked what a breastfeeding mother with signs and symptoms of COVID-19 should do. Accordingly, 1935 (61%) of the participants indicated that the mother should continue breastfeeding while taking the necessary precautions, whereas 560 (17.6%) of the participants responded that the mother should continue breastfeeding as before the COVID-19 pandemic. Conversely, 327 (10.3%) and 194 (6.1%) of the participants said the child should be separated from the mother immediately, and the child should be given substitute milk and not breastfed, respectively (Table 3).

**Table 3:** Perceptions and awareness of appropriate breastfeeding practice if a mother is suspected/confirmed with COVID-19, 2020

Perceptions and awareness of appropriate breastfeeding practice	Exposed to an advertisement about how to appropriately and safely breastfeed a baby			Propensity score analysis
	**Yes** ***n* (%)**	**No** ***n* (%)**	**Total** ***n* (%)**	**Standardized proportion difference (95% CI)**
**Total participants (*n* = 3170)**				
* What do you think a breastfeeding mother with COVID-19 symptoms should do?*				0.16 (0.12, 0.19)^**^
Continue breastfeeding	366 (23.2)	194 (12.1)	560 (17.6)	
Continue breastfeeding with precaution	982 (62.3)	953 (59.7)	1935 (61)	
Child should be given substitute milk and not breastfed	64 (4)	130 (8.1)	194 (6.1)	
Child should be separated from the mother immediately	131 (8.3)	196 (12.2)	327 (10.3)	
Do not know	33 (2)	121 (7.6)	154 (4.8)	
Total, *n* (%)	1576 (100)	1594 (100)	3170 (100)	
**Mothers (or their partners) with children < 2 years old (*n* = 1057)**				
* Do you think you/your partner will continue breastfeeding your child, if you are/she is suspected or confirmed of COVID-19?*				0.06 (0.01, 0.12)^*^
Continue breastfeeding	342 (62.7)	235 (45.8)	577 (54.5)	
Discontinue breastfeeding	137 (25.1)	174 (33.9)	311 (29.4)	
Do not know	66 (12.1)	103 (20.1)	169 (15.9)	
Total, *n* (%)	545 (100)	512 (100)	1057 (100)	

^*^P < 0.05

^**^P < 0.0001

It was found that a comparable percentage of respondents knew that a breastfeeding mother experiencing COVID-19 symptoms should continue breastfeeding with precaution in both groups of participants—those exposed to the media campaign and those who were not. The PSM analysis using a two-sample test of proportions was employed to estimate the impact of the media campaign on awareness and perceptions of the importance of continuing breastfeeding when a mother is suspected or confirmed with COVID-19. In the PSM analysis, exposure status to the media campaign was highly significantly associated with awareness and perceptions of the importance of continuing breastfeeding during the COVID-19 pandemic (PD, 0.16; 95% CI, 0.12**–**0.19; *p* < 0.0001) (Table 3).

Moreover, the awareness and perceptions of appropriate breastfeeding practices, specifically among mothers or their partners with children under two years old, were analyzed by their exposure status to the media campaign messages. Consequently, 342 (62.7%) of the mothers or their partners who had accessed the mass media adverts and 187 (46.4%) of the mothers or their partners who had not accessed the mass media campaign messages were aware of the importance of maintaining breastfeeding in case the mother is suspected or confirmed with COVID-19. Similarly, for this target interest group, the PSM analysis to assess the impact of the media campaign on awareness and perceptions of appropriate breastfeeding when a mother is suspected or confirmed with COVID-19 demonstrated statistical significance (PD, 0.06; 95% CI, 0.01**–**0.12; *p* = 0.01).

From the total of 867 participants who responded whether they had been suspected or confirmed with COVID-19, 106 (12.2%) were mothers (or their partners, in the case of male respondents) of children under the age of two who were suspected or confirmed cases at the time of the survey. Then, mothers or their partners were asked whether the child had been breastfed while following the necessary precautions. The question was styled to be responded by the participants based on their gender, “Did you (your partner) continue breastfeeding your child while taking necessary precautions?” And of them, 60 (56.6%) continued to breastfeed their child taking all the necessary precautions, such as wearing masks and maintaining personal hygiene. In contrast, 46 (43.4%) of the mothers who were suspected or confirmed with COVID-19 had discontinued breastfeeding because of concerns that the virus might be transmitted to the child and thought it would be better to cease breastfeeding until recovered.

To assess the impact of the media campaign on the breastfeeding behaviors of mothers with suspected or confirmed COVID-19 illnesses, we also compared their breastfeeding practices based on their exposure status to the media campaign messages. Accordingly, 44 (59.4%) of the 74 mothers (or their partners) who heard or seen the media advertising continued breastfeeding while taking the necessary precautions. Unfortunately, due to the small total number of suspected or confirmed breastfeeding mothers or their partners (i.e., 106), we were unable to apply the propensity matched sample analysis to estimate the impact of the media ads, specifically, on the awareness/perceptions of mothers suspected or confirmed with COVID-19.

## Discussion

Breastfeeding practices are strongly associated with mothers’ knowledge, perceptions, and beliefs [50]. Yet, the COVID-19 pandemic posed a risk by negatively influencing perceptions and attitudes about the continuation of appropriate and safe breastfeeding practices due to concerns about potential mother-to-child virus transmission, endangering existing poor child nutrition status in LMICs such as Ethiopia [8]. To address this challenge, UNICEF/Ethiopia conducted a nationwide media campaign during the COVID-19 pandemic. Although the media campaign focused on breastfeeding mothers, mothers’ partners (fathers) and the general public were also considered potential targets due to their role in influencing IYCF decisions [51, 52].

The media campaign spots were seen or heard by 51.5% of mothers or their partners with children under two years old and 49.7% of the general public. This finding could imply some practical lessons in terms of media campaign approaches used for similar media health communication efforts. Despite the use of multiple media channels to convey breastfeeding messages, it appears that the frequency of the media spots—one per day and four times per week—may be insufficient. In another similar mass media campaign implemented in Mekelle, Ethiopia, 47% of mothers were found to have been exposed to the campaign, which is comparable to our study finding, and the frequency of airing was three times per week, two times per day on television, and once per day on the radio [34]. Regardless of the number of media channels used, the frequency of the media spots is important in determining the level of exposure to the campaign as well as its effectiveness. In this respect, the saturation-based approach, i.e., high exposure to media campaign messages 6**–**12 times per day for radio spots and at least 3 times per day for TV spots, may have been better in terms of maximizing campaign reach [53].

In addition, previous similar media interventions indicated that to increase wide exposure, campaigns should include other complementary communication approaches such as inter-personal face-to-face communications and support groups [52, 54, 55]. In a mass media campaign aimed at promoting exclusive breastfeeding in Vietnam, for example, there was a greater exclusive breastfeeding behavior change in communes where the mass media campaign was implemented alongside other intervention strategies such as encouraging women to seek IYCF support from franchise centers, as well as a process of social diffusion [55].

There is no discernible disparity in the reach of the media campaign by region or other sociodemographic characteristics. This could be because the media campaign was nationwide, and the source of information and level of access to national media platforms may have been comparable across regions. According to the study findings, half of the urban and rural participants who were exposed to media spots about safe breastfeeding practices got information from television and radio, respectively. Indeed, this could spark an interesting discussion about the potential role of mass media campaigns in reducing inequalities in IYCF knowledge and practices in LMIC settings. When mass media campaigns use a combination of national and regional media platforms to offer comparable similar access to different geographic populations, it may provide an opportunity to deliver public health communication messages to a large proportion of the target audience equitably [56].

Even though we were not able to assess the effect of the media campaign on breastfeeding mothers with COVID-19 due to the small number of participant mothers suspected or confirmed with COVID-19, exposure to the media campaign was positively associated with and influenced the breastfeeding mothers’ and the general publics’ awareness and perceptions about the importance of continuing appropriate and safe breastfeeding practices. Likewise, a growing body of literature suggests that media campaigns can have a significant impact on promoting IYCF by influencing knowledge, beliefs, and practices [23, 57, 58]. Evidence-based and appropriately designed media campaigns may serve as effective behavior change communication strategies due to their advantage of reaching many target mothers at a lower cost. Studies have also indicated that theory and formative assessment-based mass media messages may have a greater impact for social and behavior change [22, 58, 59], though the need for urgent action in the context of emergencies, such as the COVID-19 pandemic in this study, may dictate public health media campaigns to be launched as early as possible, potentially jeopardizing some scientific steps. In case of an emergency, such as the COVID-19 pandemic, where the panic situation easily fuels the spread of various negative perceptions and myths, adversely affecting complex health behaviors such as IYCF, the demand for clarifying public communication messages may be prioritized while maintaining the balance between urgency and scientific social and behavior change approaches and practices, such as rapid formative research procedures.

We recognize that our study has multiple potential limitations. Due to the drawback of the mobile survey technique to include an adequate sample of COVID-19 suspected/confirmed breastfeeding mothers or their partners, we were unable to assess the impact of the intervention on breastfeeding practices among mothers with suspected/confirmed COVID-19 illnesses. The mobile survey method used also generated a sample that was outweighed mostly by males and people living in urban areas, which may limit the generalizability of the study findings. Furthermore, while PSM is a valuable statistical technique for addressing confounding in observational studies, it can only adjust for observed confounders included in the propensity score model. We suspect that there may be other confounders (e.g., education and socioeconomic status) not included in the PSM model that could be related both to the exposure and the outcome. As such, PSM may not have fully addressed selection bias, and the results should be interpreted cautiously. Finally, despite the fact that we are not aware of any other media broadcasts on IYCF during the campaign period, we do not have full confidence in the control of other media campaigns.

## Conclusions

We found that the nationwide media campaign promoting safe breastfeeding practices during the COVID-19 pandemic reached half of the target mothers and the general public. In addition, our study support that media campaigns broadcasted through a combination of multiple and a wide range of national and regional media channels can impact IYCF knowledge and practices. Future media campaigns should ensure that the intensity and frequency of media spots are appropriate, taking into account local health literacy and the media communication landscape, to achieve adequate exposure, message recall, and thereby health impact. The findings of this study may help to appreciate lessons for future mass communication efforts in Ethiopia and other similar settings that are considering using media campaigns to influence IYCF behaviors.

## Supplementary Information

Additional file 1: Figure S1. Common support groups and covariate matching in the PSM analysis

## Data Availability

The datasets generated and/or analyzed during this study include potentially identifiable personal data. As such, we are unable to make this dataset publicly available to protect the privacy of the participants and related confidentiality agreements. The corresponding author, Abel Negussie (abelnegussie@ymail.com), can be contacted to request access to anonymized data.

## Abbreviations

COVID-19: Coronavirus disease 2019
IYCF: Infant and young child feeding
IVR: Interactive voice response
LMICs: Low- and middle-income countries
PSM: Propensity score matching
PD: Proportion difference
RDD: Random digit dialing
UNICEF: United Nations Children’s Fund
