# Publisher Correction: Reach and impact of a nationwide media campaign in Ethiopia for promoting safe breastfeeding practices in the context of the COVID-19 pandemic

**DOI:** 10.1186/s44263-024-00074-1

**Published:** 2024-06-24

**Authors:** Abel Negussie, Bereket Tefera, Elyas Melaku Mazengia, Ariam Hailemariam, Ephrem Lejore, Tariku Dejene, Abiy Tefera, Ramadhani Noor, Stanley Chitekwe, Hiwot Getachew, Rachana Sharma, Eshetu Girma

**Affiliations:** 1 Department of Social and Population Health, Yirgalem Hospital Medical College, Yirgalem, Ethiopia; 2 School of Public Health, College of Medicine and Health Sciences, Dire Dawa University, Dire Dawa, Ethiopia; 3 School of Public Health, College of Health Sciences, Debre Markos University, Debre Markos, Ethiopia; 4 School of Medicine, College of Health Sciences, Addis Ababa University, Addis Ababa, Ethiopia; 5 School of Public Health, College of Medicine and Health Sciences, Hawassa University, Hawassa, Ethiopia; 6 Center for Population Studies, College of Development Studies, Addis Ababa University, Addis Ababa, Ethiopia; 7 Nutrition Section, United Nations Children’s Fund, Addis Ababa, Ethiopia; 8 Department of Global Health and Population, Harvard T. H. Chan School of Public Health, Boston, MA USA; 9 Social and Behavior Change (SBC) Section, United Nations Children’s Fund, Addis Ababa, Ethiopia; 10 School of Public Health, College of Health Sciences, Addis Ababa University, Addis Ababa, Ethiopia; 11 Ethiopian Health Education and Promotion Professionals Association (EHEPA), Addis Ababa, Ethiopia

**Publisher Correction** **:** **BMC Global Public Health 2, 37 (2024)**

**https://doi.org/10.1186/s44263-024-00065-2**

Following publication of the original article [1] it was reported that there was an error in affiliations 7 and 9 due to a typesetting mistake. The institution was incorrectly given as ‘Children’s Fund’ instead of ‘United Nations Children’s Fund’.

The correct affiliations are given in this Correction and the original article has been updated.
