# Supplementary material for: Reach and impact of a nationwide media campaign in Ethiopia for promoting safe breastfeeding practices in the context of the COVID-19 pandemic
Source: BMC Glob Public Health. 2024 Jun 10;2:37. doi: 10.1186/s44263-024-00065-2 (PMC11622923; doi:10.1186/s44263-024-00065-2)
Supplement: Supplementary file 1 — Additional file 1: Figure S1. Common support groups and covariate matching in the PSM analysis [file 44263_2024_65_MOESM1_ESM.docx]

| 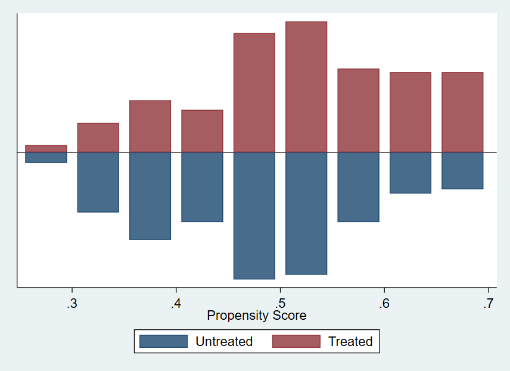 | 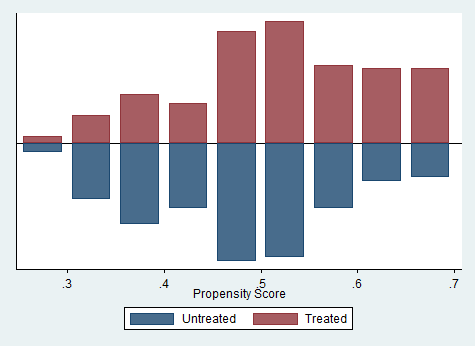 |
| --- | --- |
| Total participants – Common Support | Mothers with < 2 Years Child – Common Support |
| 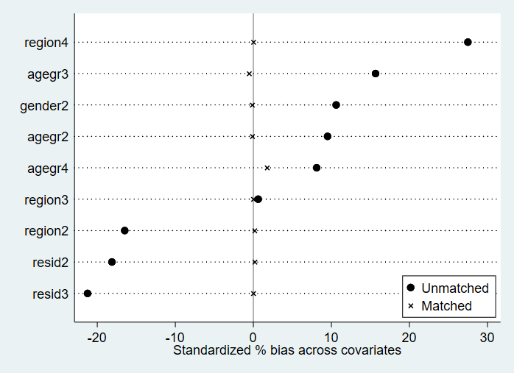 | 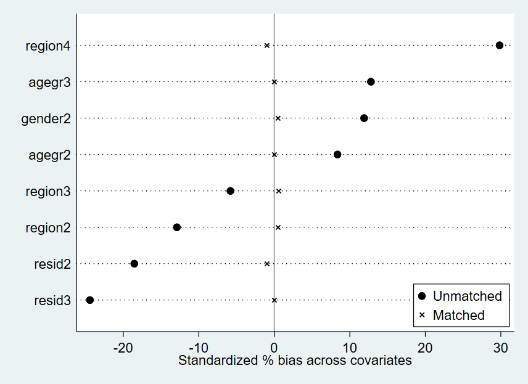 |
| Total participants – Covariate Matching | Mothers with < 2 Years Child – Covariate Matching |

**Figure S1:** Common support groups and covariate matching in the PSM analysis
